# Clinician Perspectives and Design Implications in Using Patient-Generated Health Data to Improve Mental Health Practices: Mixed Methods Study

**DOI:** 10.2196/18123

**Published:** 2020-08-07

**Authors:** Danny T Y Wu, Chen Xin, Shwetha Bindhu, Catherine Xu, Jyoti Sachdeva, Jennifer L Brown, Heekyoung Jung

**Affiliations:** 1 Department of Biomedical Informatics College of Medicine University of Cincinnati Cincinnati, OH United States; 2 Department of Pediatrics College of Medicine University of Cincinnati Cincinnati, OH United States; 3 School of Design College of Design, Architecture, Art, and Planning University of Cincinnati Cincinnati, OH United States; 4 Medical Sciences Baccalaureate Program College of Medicine University of Cincinnati Cincinnati, OH United States; 5 Department of Psychiatry and Behavioral Neuroscience College of Medicine University of Cincinnati Cincinnati, OH United States

**Keywords:** patient-generated health data, mental health, workflow, mobile application, interview

## Abstract

**Background:**

Patient-generated health data (PGHD) have been largely collected through mobile health (mHealth) apps and wearable devices. PGHD can be especially helpful in mental health, as patients’ illness history and symptom narratives are vital to developing diagnoses and treatment plans. However, the extent to which clinicians use mental health–related PGHD is unknown.

**Objective:**

A mixed methods study was conducted to understand clinicians’ perspectives on PGHD and current mental health apps. This approach uses information gathered from semistructured interviews, workflow analysis, and user-written mental health app reviews to answer the following research questions: (1) What is the current workflow of mental health practice and how are PGHD integrated into this workflow, (2) what are clinicians’ perspectives on PGHD and how do they choose mobile apps for their patients, (3) and what are the features of current mobile apps in terms of interpreting and sharing PGHD?

**Methods:**

The study consists of semistructured interviews with 12 psychiatrists and clinical psychologists from a large academic hospital. These interviews were thematically and qualitatively analyzed for common themes and workflow elements. User-posted reviews of 56 sleep and mood tracking apps were analyzed to understand app features in comparison with the information gathered from interviews.

**Results:**

The results showed that PGHD have been part of the workflow, but its integration and use are not optimized. Mental health clinicians supported the use of PGHD but had concerns regarding data reliability and accuracy. They also identified challenges in selecting suitable apps for their patients. From the app review, it was discovered that mHealth apps had limited features to support personalization and collaborative care as well as data interpretation and sharing.

**Conclusions:**

This study investigates clinicians’ perspectives on PGHD use and explored existing app features using the app review data in the mental health setting. A total of 3 design guidelines were generated: (1) improve data interpretation and sharing mechanisms, (2) consider clinical workflow and electronic health record integration, and (3) support personalized and collaborative care. More research is needed to demonstrate the best practices of PGHD use and to evaluate their effectiveness in improving patient outcomes.

## Introduction

### Background and Significance

With advances in mobile technology and the pervasive use of wearable devices, a large amount of digital health data have been generated by patients. Patient-generated health data (PGHD) refer to “health-related data created and recorded by or from patients outside of the clinical setting to help address a health concern,” as defined by the Office of the National Coordinator for Health Information Technology (ONC) [1]. According to the ONC, the adoption of PGHD can have several benefits, including, but not limited to, enhancing patient experience, alerting care teams for early intervention, and improving patient health outcomes [1]. Several projects have piloted these ideas and implemented informatics solutions to collect, use, and share PGHD [2], such as in postsurgical surveillance [3,4]. Patients seem to have positive attitudes toward PGHD and are willing to share their data with the care team to support long-term health management. Studies have shown the feasibility of using PGHD to support personalized and effective care management [5]. Using PGHD can improve data work for both clinicians and patients. A case study at a cancer rehabilitation clinic showed that the data collection became more distributed, the nurses asked more focused questions during the consultations, and the patients gradually developed competence in managing their own health [6].

Despite the potential benefits of PGHD, it currently remains limited with a focus on health history, verified surveys, and biometric activities [7]. Ultimately, PGHD should be seamlessly integrated with electronic health records (EHRs) to support the clinical decision-making process [8]. However, several challenges need to be addressed to maximize the benefits of PGHD, as many pertain to its use at the point of care. First, integrating PGHD into a clinical setting necessarily involves considering clinical workflow redesign, data management concerns, patient privacy protections, and ease of PGHD use [9]. This is especially important because clinicians spend a significant amount of time (25%-50%) on documentation tasks in their daily work [10-12]. Second, clinicians may have concerns about the impact of PGHD on reimbursement and data reliability, as PGHD are generated by patients in their daily lives and require extra effort to be consumed in clinics [13,14]. Third, PGHD use impacts the relationship between both patients and clinicians. Therefore, a collaborative model may be developed to fulfill the desire of both parties: patients’ desire to know more about their health and clinicians’ desire to have better practices [15]. With the growing interest in patient-centered health care, there have been studies that investigate patients’ motivation and attitudes toward tracking and sharing personal data [16,17]. However, the extent and methodology behind the use of PGHD and its integration into clinicians’ workflow remain unknown and, therefore, require further investigation into the demonstration of best practices.

### Objectives

To provide empirical evidence to address these challenges, this study investigated the current use of PGHD within mental health practices, with a focus on workflow, clinicians’ perspectives, and data interpretation and sharing. The main reason for choosing a mental health practice setting was that clinicians often rely on patient narratives, observations, and patient-reported outcomes (PROs) to assess the efficacy of psychiatric treatment [18]. PROs refer to “a measurement of any aspect of a patient's health status that comes directly from the patient” [19]. As the purpose of both PROs and PGHD is to collect data from the patients’ perspective, clinicians from a mental health practice are more likely to adopt and use PGHD. A total of 3 research questions arise from this: (1) What is the current workflow of mental health practice and how are PGHD integrated into this workflow, (2) what are the clinicians’ perspectives on PGHD and how do they choose a mobile-based app (mobile app) for their patients, and (3) what are the features of current mobile apps in terms of interpreting and sharing PGHD?

### Literature Review

#### PGHD

Traditionally, clinicians focus on collecting one-time snapshots of patient information in a clinical setting and making decisions based upon them, thereby losing an opportunity to create a thorough understanding of the patient’s health status [20]. In these situations, PGHD are a useful tool for continuous monitoring, especially for patients with chronic conditions that require daily management and benefit from effective tracking [21-23]. PGHD can also improve disease surveillance by more accurately assigning patients to disease categories rather than solely using national and regional data [24].

PGHD can come from multiple data sources, such as family history, medication, and physiological data sensing [25]. PGHD, along with other health data, can ultimately form a repository of patient-centered personal health records, which can then be used to store and manage PGHD [26]. Clinicians see the potential of PGHD but raise some concerns about its use, such as difficulties in summarizing PGHD patterns across different clinical specialties and concerns regarding data management, patient privacy, and ethical challenges of PGHD generation [9,27,28].

#### Opportunities and Challenges of Mobile Technology

Internet use has been growing steadily since 1990, with approximately 4 billion internet users worldwide, with 15- to 24-year-olds leading the frontier of internet adoption [29,30]. The internet has also become increasingly mobile. An unsurprising result of this trend has been increasingly sophisticated mobile technology and wearable devices, including the development of numerous mobile health (mHealth) apps, all of which have helped generate large amounts of PGHD.

Wearable devices and mHealth apps can facilitate health behavior improvements if designed with proper engagement strategies and data collection methods [31,32]. Effective mHealth app design has helped users monitor and promote positive health habits, such as physical activity and eating behaviors [33,34]. Furthermore, PGHD can help improve clinical trials’ efficiency and output [35]. However, these opportunities come with a number of challenges [2]. First, the management and interoperability of massive PGHD require standard vocabularies and data models. Consumer health vocabulary (CHV) is one such effort that has been developed since 2007 [36,37] and enhanced by several text mining–based projects [38-41]. In addition, frameworks and data models have been proposed to incorporate PGHD into EHRs [42,43]. However, very few projects use both CHV and common data models to facilitate PGHD comprehension. Indeed, to our knowledge, only one position paper proposes an interpretability-aware framework to systematically understand PGHD [44]. The second challenge is the lack of guidelines and best practices for integrating PGHD into the clinical workflow [15]. In addition, most PGHD are collected through mHealth apps, which should be carefully evaluated in a standardized manner for efficacy and health outcome improvement [45]. Finally, there are concerns about the quality and ownership of PGHD gathered by mHealth apps and wearable devices [28,46]. App developers should develop more transparent data ownership policies so that users can make informed decisions regarding their PGHD [47]. Guidelines should also be developed to ensure that high-quality data are gathered by users in their daily routines [48].

#### PGHD Use in Psychiatry

Psychiatry is a medical specialty focused on “the diagnosis, treatment, and prevention of mental, emotional, and behavioral disorders” [49]. Clinicians use a variety of data from patients to determine psychiatric diagnoses. However, these data are often collected solely in clinical settings. Since the 1960s, mental health clinicians have begun to pay more attention to patients’ personal perspectives, including health-related quality of life (HRQoL) outcomes. HRQoL is a type of PRO that includes “symptoms of disease or health condition, treatment side effects, and functional status across physical, social, and mental health life domains” [50]. PROs are derived from patients completing standardized questionnaires and cannot guarantee large-scale, continuous data collection [51,52]. However, with the help of mobile technology and wearable devices, PGHD can collect large amounts of patient health data unobtrusively and continuously. Furthermore, because psychiatry has historically depended on PROs and HRQoLs, clinicians in this field should be able to use PGHD without many conceptual barriers.

For instance, ecological momentary assessment (EMA) was proposed to assist clinical psychologists in monitoring HRQoL changes by tracking patient behaviors in real time and in their natural environment. EMA uses various data collection tools, such as written diaries and telephones, and PGHD gathered by mHealth apps and wearables can further support this approach [53]. PGHD can facilitate large-scale environmental psychiatric research in naturalistic settings and create *digital footprints* to measure patients’ health status (eg, mood and sleep) in an unobtrusive and longitudinal fashion [54].

Although studies have shown that wearable devices and mHealth apps help in the treatment of mental health by increasing awareness and giving reinforcement, such as in the study by Ng et al [55], research regarding the implementation of mobile technology into the care process is lacking. Studies have only just started to design and develop apps that focus on interface usability and workflow integration. Notably, Bauer et al [56] applied Principles for Digital Development to develop a highly usable mHealth app to support collaborative care for patients and generated 4 more principles based on user feedback. Recently, mental health studies have focused on PGHD, showing a transition to participatory and personalized medicine [57]. However, other stakeholders, such as consultants, policy makers, and vendors, should also be considered when integrating PGHD into mental health [58].

Clearly, more PGHD will be generated in the coming future through mobile technology and hold the potential to improve mental health practice, which has long relied on patient-reported data. Therefore, we follow this trend and aim to provide empirical evidence regarding the current use of PGHD in mental health practices, clinicians’ attitudes toward PGHD, and the mHealth app features and selection criteria considered by mental health practitioners.

## Methods

### Clinical Setting

This study was conducted at the Department of Psychiatry and Behavioral Neuroscience in a large academic hospital in the Midwest United States. The department is a nationally recognized leader in advancing the diagnosis and treatment of mental and behavioral disorders. The department has more than 90 faculty members, with half of them trained as psychiatrists with a medical degree (Doctor of Medicine or Doctor of Osteopathic Medicine). Our study targets were faculty members who are actively seeing patients. As there was a significant portion of faculty trained as psychologists (Doctor of Philosophy or Doctor of Psychology), our study participants included clinicians from both groups.

### Study Design

This study contains a set of semistructured interviews and an mHealth app review. The interviews collected qualitative data from both psychiatrists and clinical psychologists to understand their clinical workflow, attitudes toward PGHD, and the use and sharing of PGHD in clinics. In the app review, a set of mHealth apps was selected and reviewed systematically. The main objective of this review was to understand patients’ experiences of using mental health–related apps and their opinions around it. The review comments were also downloaded from the Google Play Store and the Apple Store using existing application programming interfaces (APIs) [59,60]. The app review data were summarized in terms of data interpretation and shared features. Data interpretation here is defined as the manner in which PGHD are collected and presented, either qualitatively or quantitatively assessed, and may be subsequently visualized. Qualitative assessment referred to users’ self-reported data on their sleep quality or mood status. Quantitative assessment, on the other hand, relied on the automatically recorded data by an app and its sensors. The clinicians’ perspectives and the app features were then synthesized to generate design recommendations for mHealth app designers and developers. This study was reviewed and approved by the institutional review board of the study site (IRB# 2018-6453).

### Participant Recruitment

A total of 12 clinicians, including 7 psychiatrists and 5 clinical psychologists, were recruited using convenience sampling and snowball sampling. Two coauthors facilitated the interview invitation to their colleagues; each participant was asked to provide a few names to reach out at the end of the interview. Table 1 shows the participant subgroups and their average professional experience (number of years). Male psychiatrists were the largest group among the participants (n=5), followed by slightly more female clinical psychologists.

**Table 1:** Participant subgroups.

Characteristics of interviewees	Male	Female	Years of experience, mean (SD)
Psychiatrist, n (%)	5 (41)	2 (17)	13.9 (6.7)
Clinical psychologist, n (%)	2 (17)	3 (25)	12.0 (10.3)
Years of experience, mean (SD)	14.5 (9.3)	11.1 (6.2)	13.1 (8.0)

### Qualitative Interviews and Analysis

Each semistructured interview was conducted by 2 members in the research team and lasted for 30 to 45 min. The interview questions were organized in 5 areas: (1) job title and responsibility, (2) clinical workflow, (3) PGHD attitudes, (4) the selection and use of mHealth apps, and (5) the use and sharing of PGHD. Although participants’ attitudes toward PGHD were specifically asked, their attitudes toward EHR integration was not prompted. The interviews were audio recorded and transcribed verbatim in 2 steps. First, the audio recordings were transcribed by the Google Cloud Speech-to-Text API. Second, the transcription drafts were reviewed by the research team to ensure high quality and deidentification of the data. The participants were coded from P01 to P12, with the first 7 participants being psychiatrists.

Interview data were analyzed in multiple rounds. Specifically, the transcriptions were coded using the Work Elements Model with a focus on actors, actions, and artifacts [61]. In the first round, a set of swim lane workflow diagrams was generated. In this swim lane chart, the columns (lanes) represent the actors, and the rectangles represent the actions. A workflow diagram was drawn for each psychiatrist. All psychiatric workflow charts were then consolidated into one workflow chart. The same process was repeated for the psychologists. It is worth noting that 3 participants (P09, P10, and P12) had a portion of the workflow that significantly deviated from others because of their job responsibilities. This reflects the variety of mental health practices. The deviation was due to our sampling methods and the relatively small sample size. These portions of workflow were excluded from the consolidated workflow because of their uniqueness.

Following the steps in thematic analysis [62], the transcriptions were analyzed to understand clinicians’ attitudes toward PGHD and the interpretation and sharing of PGHD in clinics. To ensure the coding quality, one researcher independently coded all the transcriptions and extracted themes, which were then reviewed by another researcher. The 2 researchers met and resolved any disagreements.

### Sleep and Mood App Tracking Review

A total of 31 sleep tracking apps and 25 mood tracking apps in current mobile app markets (App Store and Google Play) were selected and reviewed. Instead of developing our own search keywords, the results of 2 published systematic review papers were used [63,64], providing a list of 73 and 32 tracking apps for sleep and mood, respectively. The papers were published in 2018 and 2019. The details of the sleep and mood app selection are described in Multimedia Appendix 1.

The 2 types of mHealth apps were selected because of their significant role in tracking and understanding patients’ status and treatment effects. This was indicated through the prevalent use of these 2 types of apps by the participating psychiatrists and clinical psychologists, as shown in our interviews with them. In total, 9 of 12 participants mentioned these apps. Specifically, 3 of them reported using both sleep tracking and mood tracking mobile apps, 3 of them reported using only mood tracking apps, and 3 of them reported using only sleep tracking apps. Biometric or fitness tracking apps were also mentioned but less frequently; therefore, they were not our investigation focus.

The information of the selected apps was extracted and organized using a spreadsheet in the following 10 columns: (1) app type (either sleep or mood), (2) app ID, (3) source (Apple or Google), (4) app name, (5) rating, (6) advantages from review, (7) hindrance from review, (8) data interpretation features, (9) data sharing features, and (10) review quotes. The app review data were extracted independently by 2 researchers, one of whom reviewed sleep apps and the other who reviewed mood apps. The extracted data were then reviewed by another researcher for quality checks. Information was collected solely from users' comments. Advantages and hindrances were defined as qualities that users predominantly classified as benefiting the user experience or detracting from it, respectively. The details regarding app sharing capabilities (such as social media and exports to other file types) and methods of data display and interpretation were also included. Selected quotes were compiled from users who offered a holistic perspective on the app. It is worth noting that app review data mostly reflected end user experience and highlighted general user satisfaction or complaints with the apps, complementing our interviews with the clinicians.

## Results

### Qualitative Analysis: Clinical Workflow

#### Consolidated Workflow and PGHD Use

**Figure 1:**
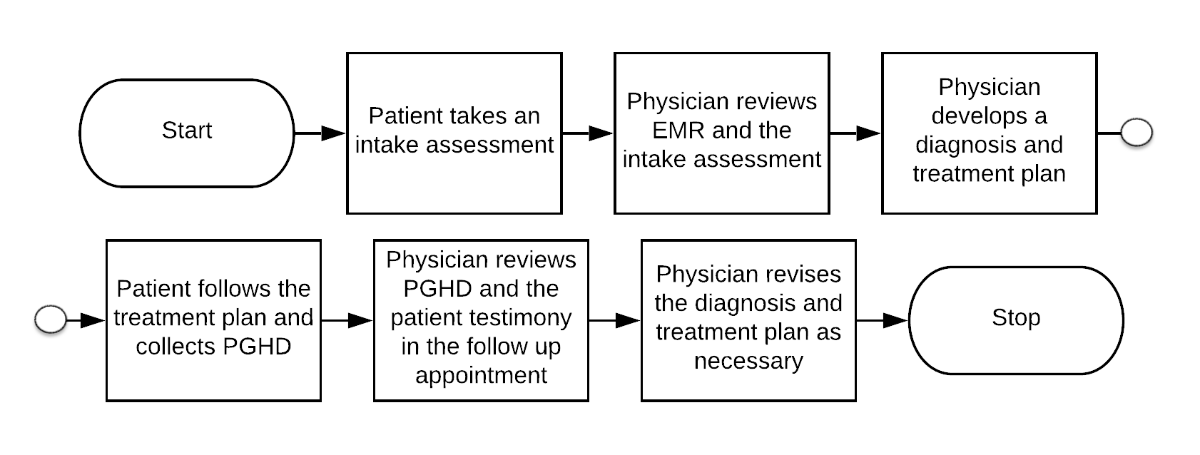
Simplified consolidate workflow diagram of psychiatrists and psychologists based on semistructured interviews. EMR: electronic medical record; PGHD: patient-generated health data.

Figure 1 shows a simplified version of the consolidated workflow diagram, which includes step-by-step movement from a patient taking an initial assessment, development of diagnosis and treatment plan, meeting with a physician, the influence of PGHD, and subsequent adjustments in the diagnosis and treatment plan. The detailed workflow diagram is included in Multimedia Appendix 2.

As shown in Figure 1, a typical clinician workflow in mental health started from gathering patient information and PROs using an intake assessment. Mental health clinicians then reviewed the form along with EHR information, if any, and conducted a clinical interview to establish psychiatric diagnoses and a corresponding treatment plan. Next, patients participated in the treatment (eg, practice assignments and collection of PGHD), following their clinicians’ requirements. The ways to collect PGHD varied depending on the clinicians’ preferences and patient situations. Next, in subsequent clinical encounters, clinicians reviewed the PGHD and assessed the changes in their patients’ symptoms and functioning since the previous session. Clinicians may revise the treatment plan based on the updated patient health status.

Many participants do not consider themselves as the sole decision maker in developing the treatment plan. Instead, they worked with patients to align their goals with the treatment. Homework is a common term used by clinicians to discuss patients’ efforts to improve their mental health status between clinic visits. PROs and PGHD can assist patients in their homework, demonstrate their achievement, and provide information to clinicians to make evidence-based decisions on the treatment plan and assess treatment progress. However, participants reported no standardized way to collect and manage PGHD.

Although collecting PROs and PGHD has been part of mental health practices, its use is not optimized. Taking the intake forms as an example, one participant talked about troubles using survey scales when patients wait in the waiting room. This suboptimal data collection may slow down the clinics and reduce the efficiency and quality of care:

As I mentioned the scales aren't always filled out and they may not have enough time to fill out the scales if they're call[ed] back right away.

P06

Sometimes, PROs can be ambiguous and confusing and require more data to understand patients’ health status change and nuanced differences. In this case, PGHD can be complementary and provide more detailed behavioral data to inform the shared clinical decisions on the treatment plans. One participant explained how PROs may be confusing:

So, for example, like number three on the PHQ9, in one item, it's assessing “trouble falling asleep,” “staying asleep” or “sleeping too much,” which could mean very different things in terms of planning to do for treatment. And so, I will always ask a follow-up and then I will manually circle the ones that applied for the patient.

P10

#### Workflow Comparison Between Psychiatrists and Clinical Psychologists

All participants followed the simplified consolidated workflow in their practices. However, there were some noticeable differences between psychiatrists and clinical psychologists. Psychiatric appointments, in general, were shorter (30 min), followed by appointments with clinical psychologists (45-60 min). As psychiatrists can prescribe medication, they check medication use and effects in every patient visit. Psychiatrists also conduct psychotherapy and value the therapeutic relationship between patients and themselves. On the other hand, clinical psychologists cannot prescribe medication, so they focus solely on conducting psychological assessments and delivering psychotherapy. Therefore, the workflow for clinical psychologists can be very dynamic and conversational. Clinical psychologists pay significant attention to patients’ narratives of experience to understand their unique health status and changes. The workflow would be more standardized, however, if a clinical psychologist only focuses on psychological assessments, for example, a cognitive evaluation, as the assessments have a validated procedure to follow and tools to use.

### Qualitative Analysis: Clinician Perspectives

#### Dual Attitudes Toward PGHD

All participants had worked with their patients to track their sleep and/or mood behaviors through an mHealth app and/or a wearable device. Overall, 6 participants held a dual attitude toward PGHD use. On the one hand, clinicians had seen the potential of PGHD and looked forward to taking advantage of them, especially the ability to track their patients’ activities between consultations better:

There is a long history of using mood scales, potentially longitudinal mood scales, basic tracking charts for depression, bipolar disorder—can be really helpful, also can be somewhat tedious. And there are some apps now that do that very well...”

P05

On the other hand, some concerns were raised about data validity and reliability. The participants were being cautious because they identified the need for reliable PGHD to inform evidence-based treatment implementation and evaluation:

When you're saying hard data from a device that measures sleep, I would need to know for myself how it's measuring sleep... I think I would tend to question the specificity and accuracy of those for actual sleep... I find that patient self-reports of sleep are unreliable.

P04

#### Concerns About Integrating PGHD Into Workflow and EHR

Clinicians’ concerns about integrating PGHD into workflow and EHR systems were a recurring theme in the interviews. One participant indicated that there was no app that seamlessly integrates its data into EHR:

So... the way things work now [is] very much sort of pen and paper. You show up at a doctor's office. They get handed some of these screeners and somebody must manually enter it into Epic, which is kind of a pain. We would love to be able to send patients a MyChart message or something and say fill this out and send it back to us and have it automatically go into a flow sheet in Epic that we then track over time with the patient. That would be amazing.

P03

A total of 7 participants preferred using paper-based PGHD in their practice because of the patients’ preference or the lack of data sharing mechanisms in the apps. In this case, the data flow is deemed indirect. Clinicians would review the PGHD in the session and put the interpretations in the clinical notes, which can slow down the clinical workflow. Generating data visualization of PGHD to facilitate the interpretation of the data seems to be a preferable method for both clinicians and patients:

We also graph patient data over time. I do this with all my patients... It would take 20 minutes of the patient session—patient wouldn’t get care for that 20 minutes and it’s literally I’ll have a calculator out, an actual calculator, my graphing calculator, and I’ll sit there and calculate the data and then manually make the graph. So having programs built in that aggregate data and automatically populate graphs are great; and patients like to see the visual, the graph—they do, overall. And generally it’s good discussion even if the graph is not great in terms of what it’s explaining.

P10

Manually transcribing data into electronic medical records is not only time consuming but also interferes with the patient-clinician interaction because the clinicians are distracted by the data entry tasks on their computer. Common concerns found included the difficulties of having to face the computer and type in data during sessions:

I'm put[ting] in the data in the computer and looking at the questionnaires and if there was a different way of doing that, you know, it would actually make me take my life away from the computer screen and interact with my patient.

P01

Lack of Information for App Selection

In addition to the challenges in using data from mHealth apps, it can be challenging to identify what apps to use in the first place. In all, 3 participants found that it was difficult to find an app that met all the requirements. Others sometimes found useful apps through patient recommendations. The following 2 quotes exemplify this situation:

I've not been able to figure out an app that I could actually use [and] that was specific to be able to individualized for patients; that's been a challenge.

P11

There are lots of great apps out there for sure...and sometimes they'll bring stuff to me that I'm unaware of.

P08

When asked about using apps for collecting data, some clinicians responded that they did not know that mobile apps could perform certain data tracking tasks:

So, we look at this daily measure sheet, which would be perfect for an app that looks at what their moods have been, what kind of sleep they had.

P11

In contrast, the app review (see details given in the section *Sleep and Mood Tracking App Reviews*) showed that mood apps in the current market can offer certain functions, which means there is a lack of information to increase app awareness and support app selection process, especially for clinicians. This suggests that an app recommendation system for the clinician to use would be very helpful.

#### Limited App Features to Support Personalization and Collaborative Care

One factor contributing to the difficulty of finding the right app is the variety in patients’ health statuses and conditions. Clinicians would prefer to personalize treatment and collect data in a personalized manner. However, current apps were not designed with features to support personalization in data collection. One participant elaborated that:

A psychiatric exam, ugh, depends on a lot offactors actually...Let's say I have a 60-year-old sort of borderline intellectual functioning person, maybe 8th grade education with never seen a psychiatrist and coming in first time... So that exam...maybe take more time there, more explanation, more education, more time to elicit... I may have to offer something, a different questionnaire other than the standard four to five that I sent to every patient.

P01

Another type of feature that current apps may be missing is to support collaborative care. It is beneficial to support collaborative care so that patients can be more engaged in their health and take control of their care, and clinicians can create a treatment plan that is most suitable and effective for the patient’s situation. One clinician further talked about the difficulty in finding an app in current app markets to address his needs of data collection, although they do have data collection features:

The app would have to collect data on several different domains like nutrition, like sleep, like physical activity, and then maybe sampling mood multiple times a day. So there are apps that look at the individual factors, but I don't know, I'm not aware there may be an app [that can offer all above].

P09

### Sleep and Mood Tracking App Reviews

#### Statistical Summary

Table 2 shows the statistical summary of the selected apps. The data were retrieved on September 8, 2019. The average number of reviews was around 400, and the average user rating was around 4.0 for both sleep and mood tracking apps.

**Table 2:** Statistical summary of the selected sleep and mood tracking apps.

App characteristics	Sleep	Mood
Number of apps, n (%)	31 (55)	25 (45)
Number of reviews, mean (SD)	346.1 (896.8)	387.8 (912.5)
User rating, mean (SD)	3.88 (0.78)	3.99 (0.54)
Had a data interpretation feature, n (%)	31 (55)	21 (38)
Had a data sharing feature, n (%)	18 (32)	16 (29)

#### Data Interpretation and Sharing Features

The data interpretation, which includes collection and visualization, and sharing features in the selected apps varied. All 31 sleep tracking apps offered at least one feature for data interpretation, including sleep quantity statistics (n=27) and sleep quality analysis (n=5). Of the 31 sleep tracking apps, 18 (58%) support data sharing in various means, including direct sharing with other people (n=8) and integration with other apps (n=7).

The major source of data interpretation is through collecting statistics, through recording several days' worth of accumulated data points. These data points are either manually inputted by users or automatically recorded by the app. Overall, 5 apps also offer qualitative assessments of sleep patterns by presenting the sleep cycles that users experience, recording sound files, and/or providing descriptive sleep analyses.

Many sleep tracking apps also support sharing data. This is usually done by exporting and downloading the data as a comma-separated values (CSV), PDF, etc file (N=8) or via integration with an alternate app, such as Apple Health (n=7). Other sources include social media (n=3) and email (n=4). Many apps also support multiple forms of sharing.

Many users find accurate sleep tracking to be helpful in improving their sleep quality and daily life:

I love this app. Pleasing to the eye and so many great features! I love that it keeps stats on your sleep cycles and can be set to accommodate how your sleep may be affected by working out, caffeine, or other factors.

On the other hand, common complaints of sleep apps included inaccurate tracking, failures in data collection, and difficulty in use due to technical issues. For example, one user expressed frustration in inaccurate tracking:

This app has been super frustrating. I use it while wearing my watch and it has recorded me in a deep sleep while I was making food.

The analysis of mood tracking apps shows similar results. Of the 25 mood tracking apps, 21 (84%) offered either a qualitative (n=4) and/or quantitative (n=22) data interpretation feature. Eight mood apps had more than one mode of quantitative data interpretation. Of the 25 mood tracking apps, 16 (64%) had data sharing capabilities, mainly through direct sharing with other users (n=13) or social media (n=5). Three mood apps had more than one mode of data sharing.

Many apps offer multiple means of data interpretation. The forms of qualitative data interpretation include monthly reports of the moods logged. Forms of quantitative data interpretation include daily, weekly, or monthly graphs or charts of the frequency of moods logged by the user. The modes of sharing data are exporting the data as a spreadsheet, CSV, PDF, etc file (n=6); via email (n=3); or via cloud sharing (n=3).

Many users find that the ability of the apps to track and reflect on one’s mood trends was very helpful:

I am not exaggerating to say this app has been life changing. This app allows me to customize the settings so I can simultaneously track my moods, medications, activity level, wellness tools, triggers and alternative treatments. All of this info is so important. Daily logging with an easy interface means I can track LOTS OF DATA in an organized way. The visual graphs show the relationships between these various data points. The info gathered is a valuable tool in my wellness and recovery.

The most common complaint was the limited options of mood offered, which caused difficulties for users in tracking their real mood, as indicated in the following review comments:

More moods. Exactly the kind of app I need. I just wish there were more moods like: anxious, on edge, tearful, sensitive, irritable, exhausted.

With both mood and sleep apps, the primary method of data visualization was through graphs and charts (n=37). Sleep apps, which recorded quantitative data more frequently than qualitative data, used statistics (such as the length of time slept) to present graphical summaries of sleep over time. These graphs were then analyzed to show the quality of sleep over time, track trends, inconsistencies, and improvements. Similarly, mood apps also used graphical representations to present data summaries. However, these apps typically used qualitative data entries (such as recorded moods on certain days) to provide graphs and charts that showed the number of times certain moods were recorded and how moods fluctuated over the course of weeks or months. The popular usage of graphs across both categories suggests that regardless of the nature of data collection (either quantitative or qualitative), users prefer apps that visualize data through digestible and succinct representations that make trends identifiable and trackable over time.

## Discussion

### Principal Findings

We conducted a mixed methods study with 12 clinicians in mental health practice to understand their perspectives on PGHD and the current use and to share features of the mHealth apps on the market. The results show that mental health clinicians had a dual attitude toward PGHD. The advantages and concerns of PGHD use were aligned with those in the literature. It is not surprising that mental health clinicians have seen the potential of PGHD because they have been largely relying on PROs to develop treatment plans [18]. Our results also confirmed that mental health clinicians have concerns about data validity and reliability similar to clinicians in other specialties [13,14]. Although mental health practices have started to use PGHD, their use has not been optimized in clinical workflow and integrated into EHR. However, limited PGHD integration with EHRs may not be totally native. Clinicians who have concerns with data reliability and ability may prefer to review PGHD before putting them into EHR, rather than including them directly from mHealth apps and/or wearables. Moreover, our findings revealed that there are different ways to make use of PGHD without integrating it into EHR systems, such as using it to check patient conditions and homework in between clinical sessions. However, personalized data tracking and visualization are critical factors in the successful use of PGHD for both patients and clinicians.

In addition to using PGHD in clinics, we found that mental health clinicians may have a hard time finding the right mHealth apps for their patients to collect PGHD in the first place. There was a lack of information to help them choose the most suitable apps for their patients to use. Part of the reason is that each patient has a unique mental health status and condition, and mHealth apps do not support much personalization. Moreover, most of the mHealth apps were patient centered but may not support collaborative care. As clinicians and patients frequently make shared decisions for the treatment plan, mHealth apps without features to support collaborative care could reduce clinicians' willingness to adopt them or introduce barriers in clinical workflow. In addition, our review on sleep and mood tracking apps confirmed that the current mHealth apps on the market had limited features in data interpretation (eg, visualization) and limited mechanism to share PGHD with other people and EHR systems.

### Design Implications

#### Improve Data Interpretation and Sharing Mechanisms

Current mood or sleep tracking apps are focused on collecting PGHD in a patient-centered manner, which is a critical first step. However, to maximize the value of PGHD, these apps should improve their mechanisms in data interpretation and sharing. Specifically, data visualization can be a viable way to help both mental health clinicians and patients interpret much PGHD and identify patterns and trends regardless of their integration into EHR. On the other hand, mHealth apps should enable data sharing mechanisms with different parties, including, but not limited to, clinicians, families, friends, and other practitioners, as this was a concern noted by both clinicians during interviews and users in the app review [58]. It is worth emphasizing the importance of information confidentiality when designing a sharing mechanism. Psychology clinics are considered as a *safe bed* for patients to discuss their mental health status and conditions with clinicians. Hence, data sharing mechanisms should not be one-size-fit-all; they should be designed to allow patients to select which part of PGHD to share and how to share to keep highest data confidentiality based on their psychiatric conditions.

#### Consider Clinical Workflow and EHR Integration

Technology-enabled clinical data capture and documentation should consider the clinical workflow [65]. Similarly, we suggest that mHealth apps designed to gather PGHD should consider clinical workflow to improve the quality of patient experience. Although PROs and PGHD have been used in mental health clinics, there are no guidelines for data collection and use. It would be beneficial to conduct observational workflow analysis, such as time and motion studies, to understand when and where PGHD are used and identified bottlenecks. In fact, it is common to conduct such workflow analysis to design and develop any health information technology that will be used in clinics (eg, clinical decision support tools) [66].

On the other hand, mHealth apps should also consider EHR integration. We have discussed the importance of sharing PGHD with various parties in a way to protect information confidentiality. PGHD may be shared with clinicians directly through EHR integration or indirectly through data summarization and electronic reports. We have also seen a working example in our interviews to integrate PGHD with EHR using a Research Electronic Data Capture database [67]. As clinicians may have concerns on data reliability and accuracy, having an indirect EHR integration (eg, a dashboard or PDF report demonstrating patterns in PGHD for review) may be a viable way to reduce clinicians’ concerns and increase their PGHD adoption in clinics. As many apps are coming into the market and are also going obsolete at a fast pace, a standardized data management and export system could be proposed to better integrate PGHD into clinical practice regardless of specific kinds of apps.

#### Support Personalized and Collaborative Care

Our results showed that mHealth apps must support both personalization in data collection and collaboration between patients and clinicians during clinics. In terms of personalization, as each patient has a unique mental health status and conditions in various social contexts, it may be difficult to find an app that covers all kinds of needs of PGHD collection. There are 2 ways to approach this issue from a design perspective. First, mHealth apps should maximize their ability to personalize data collection methods to fit different patient needs. Participatory design methods may be helpful in identifying such needs and incorporating them into app features. Second, as clinicians do not always have the information of some existing apps that are potentially useful, an app recommendation system may be developed to assist clinicians in choosing which apps to use to collect PGHD. Currently, some clinicians rely on patients’ recommendations. This app recommendation system may be maintained by both clinicians and patients.

In addition, mHealth apps should be designed to support collaborative care. It is critical to ensure that PGHD are used effectively and efficiently during clinics. Ethnographic observations may be needed to systematically document the behaviors and interactions between patients and clinicians. The results can help researchers better understand the role that PGHD and mHealth apps play during clinic visits and generate guidelines to redesign mHealth apps and improve the use of PGHD to support shared decision-making and collaborative care.

### Limitations

This study has a few limitations. First, it was conducted in one institution using convenience sampling to recruit participants, thereby limiting generalizability. Second, the study only included clinicians, whereas patients’ perspectives were only indirectly approached through our research on app reviews. However, we believe that the clinician perspective on PGHD was sufficiently understood because we continued recruiting the participants until the data reached saturation. Moreover, we compared the interview data with the app review data, which were complementary, to generate the design implications. Third, the app review was conducted using a snapshot of mHealth apps investigated in the past 2 years. The apps may have since then been updated, resulting in fluctuating ratings and different features. However, the efficacy of the app review remains the same, as it helps identify the keep features that users look for in mHealth apps and provides sufficient data to inform app designers. Finally, clinicians can have several subspecialties in mental health, which affects their workflow and how they use PGHD. We were not able to recruit a diverse sample to include all the opinions from the mental health clinicians. However, this study focused on the common workflow components in mental health practices and served as a pilot study to understand clinician perspectives.

### Future Directions

We will continue investigating the best practices for using PGHD at the point of care, considering clinical workflow and developing informatics solutions to facilitate the development of a collaborative model to make sense of PGHD to inform shared decisions. The PGHD here will not be limited to data gathered from mHealth apps and wearable devices. They can include data from social media (eg, Twitter and forums) to synthesize more information about patients’ opinions on their health [68]. Interactive data visualization may be a viable way to achieve the common goals of clinicians and patients. Moreover, we will pay specific attention to the integration between PGHD and EHR and further develop clinical decision support tools with machine intelligence to use this new and valuable dataset to improve patient outcomes.

### Conclusions

This study demonstrated the clinicians’ perspectives on PGHD and the current features of mHealth apps. The results showed that PGHD have been used in mental health practices but in a suboptimal way without guidelines. Clinicians look forward to the potential benefit of using PGHD but have dual attitudes toward PGHD. That is, clinicians see the potential of PGHD but hesitate to embrace them mainly because of data validity and reliability concerns. Other concerns about workflow and EHR integration exist prevalently. Moreover, clinicians experienced challenges in selecting suitable apps for their patients, partly because of the limited features of mHealth apps in supporting personalized and collaborative care. We identified 3 design implications: (1) improve data interpretation and sharing mechanisms, (2) consider clinical workflow and EHR integration, and (3) support personalized and collaborative care. We will continue our research with a focus on designing and developing informatics solutions to demonstrate the best practices of PGHD use and evaluate their effectiveness in improving patient outcomes.

## Appendix

Multimedia Appendix 1 Sleep and mood app selection process.

Multimedia Appendix 2 Detailed consolidated workflow diagram.

## Abbreviations

API: application programming interface
CHV: consumer health vocabulary
CSV: comma-separated values
EHR: electronic health record
EMA: ecological momentary assessment
HRQoL: health-related quality of life
mHealth: mobile health
ONC: Office of the National Coordinator for Health Information Technology
PGHD: patient-generated health data
PRO: patient-reported outcome
