# Supplementary material for: Clinician Perspectives and Design Implications in Using Patient-Generated Health Data to Improve Mental Health Practices: Mixed Methods Study
Source: JMIR Form Res. 2020 Aug 7;4(8):e18123. doi: 10.2196/18123 (PMC7442947; doi:10.2196/18123)
Supplement: Multimedia Appendix 1 [file formative_v4i8e18123_app1.docx]

## Multimedia Appendix 1. Sleep and Mood App Selection Process.

The app selection process was designed in a systematic manner as shown in the following figure. Arrows indicate transition numbers from sleep and moods apps assessed to total number of apps retrieved, total number of apps included, and total number of apps excluded.

**
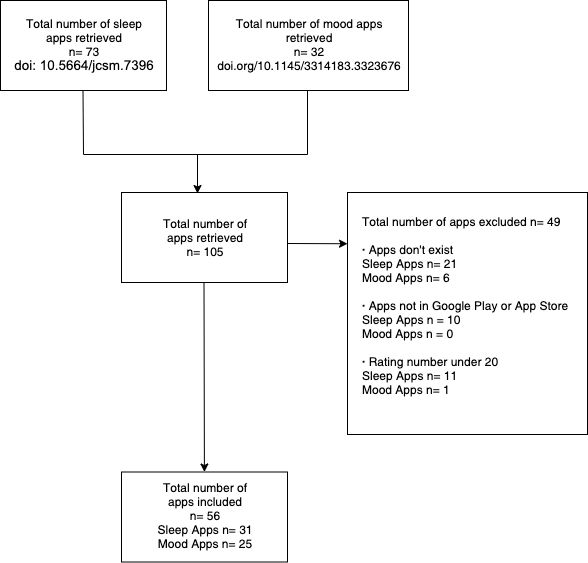
**

Instead of developing our own search keywords, the results of two published systematic review papers were utilized,[56,57] providing a list of 73 and 32 tracking apps for sleep and mood, respectively. The papers were published in 2018 and 2019. The apps were determined by the authors in the previous studies through systematic searches across different app stores using keywords related to sleep and mood tracking and management. The apps were filtered in the present study according to criteria, which included the number of ratings, main language, availability on app markets, and main purpose (which had to be either sleep or mood tracking), and, finally, by reviewers who resolved any discrepancies or conflicts. The app identifiers (App ID) of these tracking apps were provided by the corresponding authors of the papers. The detailed information of the 105 apps, including number of reviews, review rating scores, and review comments, were collected using the identifier of each app published in the papers. Out of the 105 apps, 49 were removed due to “No existence” (N=27), “Not in Google/Apple market” (N=10), and “Number of ratings less than 20” (N=12), leading to 56 apps for the analysis. Apps were excluded for having less than 20 ratings to minimize outlier bias and to ensure that the selected apps had enough comments to offer a thorough understanding of app usage.
