# Supplementary material for: Clinician Perspectives and Design Implications in Using Patient-Generated Health Data to Improve Mental Health Practices: Mixed Methods Study
Source: JMIR Form Res. 2020 Aug 7;4(8):e18123. doi: 10.2196/18123 (PMC7442947; doi:10.2196/18123)

## Multimedia Appendix 2. Detailed Consolidated Workflow Diagram.

The following figure shows detailed consolidated diagram of psychiatrists’ and psychologists’ workflow derived from the interviews. This expanded workflow demonstrates a multiple step process that varies depending on status of patient and purpose of visit, whether to diagnose or monitor treatment, as well as the involvement of other figures in the clinic besides the psychiatrist/psychologist.


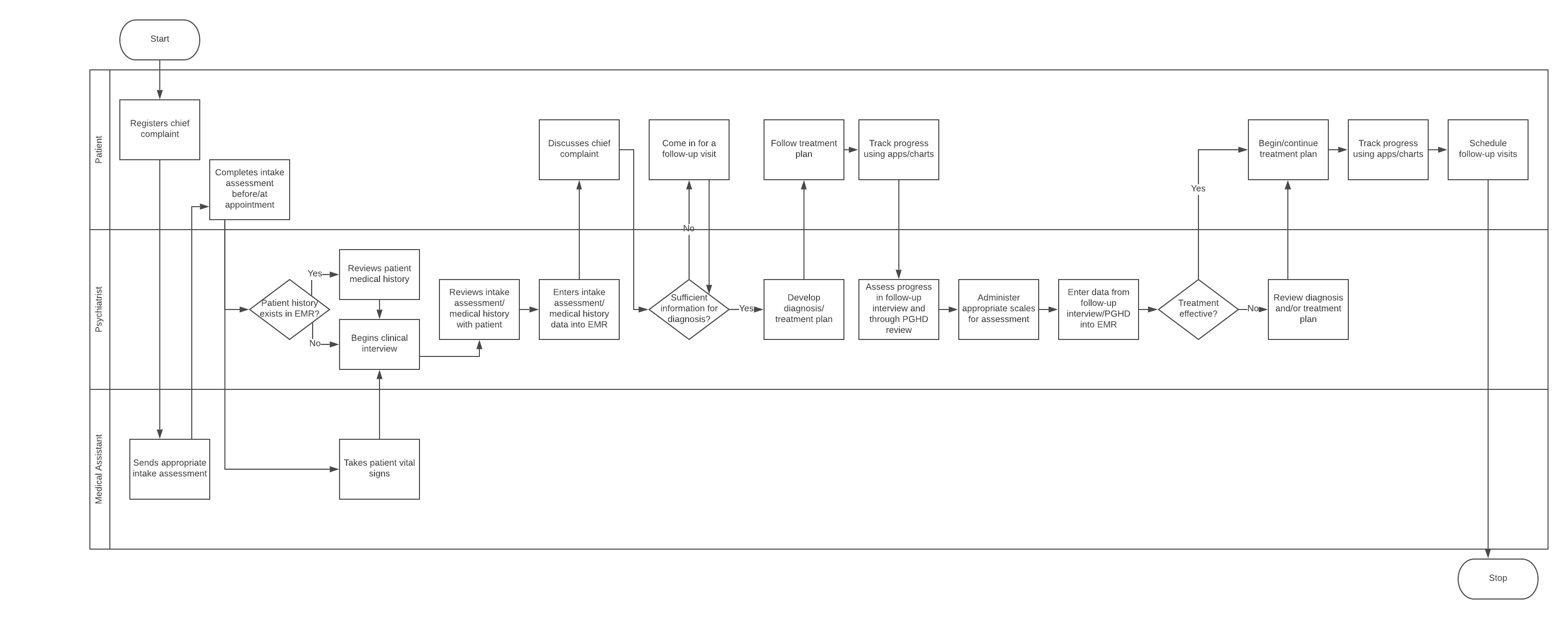

Supplement: Multimedia Appendix 2 [file formative_v4i8e18123_app2.docx]
